# The outcome of pregnancies after bariatric surgery: an observational study of pregnancies during 2004–2016 in Finland

**DOI:** 10.1007/s00404-023-06935-8

**Published:** 2023-01-26

**Authors:** Kaukonen Sesilia, Pajula Susanna, Koljonen Virve, Gissler Mika, Ulander Veli-Matti, Kaijomaa Marja

**Affiliations:** 1 Department of Obstetrics and Gynecology, Helsinki University Hospital and University of Helsinki, Haartmaninkatu 2, 00290 Helsinki, Finland; 2 Department of Plastic and General Surgery, Turku University Hospital, Turku, Finland; 3 Department of Plastic Surgery, University of Helsinki and Helsinki University Hospital, Helsinki, Finland; 4 Information Services Department, Finnish Institute for Health and Welfare, Helsinki, Finland; 5 Department of Obstetrics and Gynecology, Helsinki University Hospital and University of Helsinki, Helsinki, Finland; 6 Region Stockholm, Academic Primary Health Care Centre, Stockholm, Sweden; 7 Department of Molecular Medicine and Surgery, Karolinska Institute, Stockholm, Sweden

**Keywords:** High-risk pregnancy, Morbid obesity, Bariatric surgery, Delivery, Caesarean

## Abstract

**Purpose:**

Overweight and obesity are major risk factors for perinatal morbidity and mortality, and the need for bariatric surgery (BS) among fertile-aged women is increasing. This study evaluates the outcome of post-BS pregnancies and deliveries.

**Methods:**

All 20–45-year-old patients delivering between 2004 and 2016 in Finland were included. Patients with previous BS were identified from the hospital discharge register, and the medical birth register was queried for data on pregnancies, deliveries, and perinatal outcomes. The data were matched using personal identification codes, and the outcomes of women with previous BS were compared with those of other pregnancies.

**Results:**

Women with previous BS (*n* = 314) constituted the bariatric group. When compared with the non-bariatric group (*n* = 750,019), they were older (*p* < 0.001), heavier (*p* < 0.001) and had more previous pregnancies (*p* < 0.001). The overall incidence of pregnancy-induced hypertension (*p* = 0.002), gestational diabetes (GDM) (*p* = 0.018), pre-term contractions (*p* = 0.023), pre-term delivery (*p* = 0.003), labour induction (*p* < 0.001), planned (*p* = 0.001) and unplanned (*p* = 0.036) caesarean sections and low birthweight infants (*p* < 0.001) were significantly higher in the bariatric group. When compared with body mass index–specific categories, the main outcomes were increased incidence of GDM and small for gestational age (SGA) newborns in the bariatric group.

**Conclusion:**

BS can be considered a safe and advisable treatment for obesity among fertile-aged women. The pregnancy outcome is associated with post-BS weight, but the risk for GDM and small for gestational-age newborns is increased.

## What does this study add to clinical work

**Table:** 

Bariatric surgery is a safe and advisable treatment for severe obesity among fertile age women and the prognosis of pregnancy is highly associated with the achieved weight loss. The risk for hypertensive disorders is comparable with BMI-matched controls, but the risk for GDM and small for gestational age newborns is increased after bariatric surgery.	

## Introduction

The prevalence of overweight and obese women of childbearing age is higher than ever [1]. In 2019, 42% of parturients in Finland were overweight (body mass index (BMI) ≥ 25 kg/m^2^), and 17% were considered obese (BMI ≥ 30 kg/m^2^) [2].

Overweight and obesity are major risk factors for perinatal morbidity and mortality, increasing the risk of adverse pregnancy and delivery outcomes. They also impair fertility and affect the health of the unborn child [3].

The most effective treatment for severe obesity is bariatric surgery (BS) [4]. Bariatric procedures can be restrictive, such as sleeve gastrectomy and gastric banding or malabsorptive, such as biliopancreatic diversion. Also, combination procedures such as gastric bypass, are used. The laparoscopic sleeve gastrectomy has become the most frequently performed bariatric procedure in the world [5].

In Finland, around 1000 patients undergo BS every year [6]. Since also women of reproductive age undergo BS [7], it is crucial to identify the risks and benefits that this surgery has concerning fertility, future pregnancies, and deliveries. Even though previous studies have reported overall improved pregnancy outcomes after BS [8–10], an increased risk for pre-term delivery [8] and small for gestational age (SGA) [10] neonates have been reported.

In this study, we aim to evaluate the overall effect of BS on pregnancy, delivery, and neonatal outcome by using national register-based data on women with pregnancy after BS.

## Methods

The Helsinki University Hospital review board approved the study and its plan. Information on women with previous BS was obtained from the Finnish Institute of Health and Welfare, and its medical birth register was queried for data on pregnancies, deliveries, and neonatal outcomes. The data were merged using a personal identification number. Ethics committee approval was not required.

All pregnancies and deliveries in Finland between 2004 and 2016 were included. The data concerning maternal and pregnancy characteristics (age, smoking, parity, miscarriages, in vitro fertilisation (IVF)) and pregnancy-associated complications (pregnancy-induced hypertension (PIH), pre-eclampsia, pre-term contraction and gestational diabetes (GDM)) were analysed. Onset, type, and gestation at delivery, as well as the neonatal outcome (SGA, large for gestational age (LGA), five-minute Apgar points), were recorded. In cases of multiple pregnancies, the outcomes of A-foetuses were included in the study. The full data concerning the study period were available in 2018.

Pregnancies with previous BS were analysed, with all other study period pregnancies used as a reference group. To study more individual outcomes after BS, we compared the outcomes in four BMI-specific groups: BMI ≤ 24.9 kg/m^2^ (normal weight), 25.0–29.9 kg/m^2^ (overweight), 30.0–34.9 kg/m^2^ (obese class I) and ≥ 35.0 kg/m^2^ (obese class II).

Statistical analyses were performed using SAS version 9.4 software (SAS Institute, Cary, NC, USA), and *p* < 0.05 was considered statistically significant. Continuous and categorical variables were compared using the Mann–Whitney *U* test and the *X*^2^ test or Fisher’s exact test, respectively.

## Results

Altogether, 314 of 750,333 women (0.04%) had undergone BS before pregnancy and formed the bariatric group. The remaining 750,019 women (99.96%) formed the non-bariatric group.

**Fig. 1:**
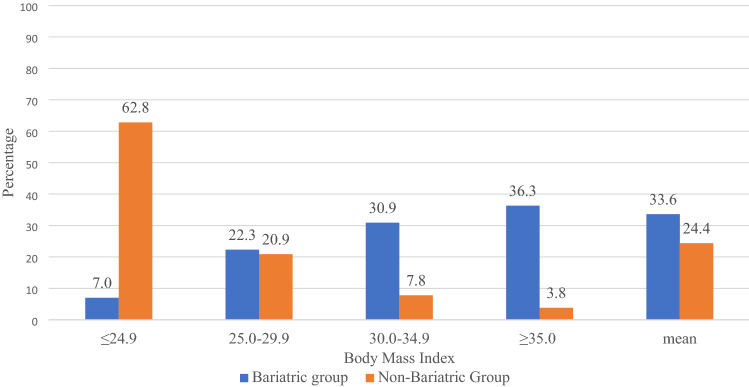
Distribution of weight in the study groups

Compared with 62.8% of women in the non-bariatric group, only 7.0% of women in the bariatric group were normal weight (BMI ≤ 24.9 kg/m^2^) at the beginning of pregnancy. In the obese classes I and II, the proportion of overweight and obese women was 22.3 and 67.2%, compared with 20.9 and 11.6% in the non-bariatric group, respectively (Fig. 1).

An overall comparison of the groups showed that women in the bariatric group were older (33.9 vs 30.2 years; *p* < 0.001), had a higher BMI (33.6 vs 24.4 kg/m^2^; *p* < 0.001) and smoked more during pregnancy (25.8 vs 15.0%; *p* < 0.001). The incidence of primiparity was lower (40.1 vs 41.7%; *p* < 0.001), and that of multiparity (13.7 vs 9.9%; *p* < 0.001), miscarriages (30.3 vs 21.3%; *p* < 0.001) and IVF pregnancies (7.0 vs 2.4%; *p* < 0.001) higher, after BS. The number of multiple births was equal (1.6 vs 1.4%; *p* = 0.811).

The incidence of PIH (*p* = 0.002), GDM (*p* = 0.018), pre-term contractions (*p* = 0.023), pre-term delivery (*p* = 0.003), labour induction (*p* < 0.001), planned (*p* < 0.001) and unplanned (*p* = 0.036) caesarean sections (CS) were all significantly more common in the bariatric group. No difference was detected in the incidence of pre-eclampsia (*p* = 0.618), insulin treatment for GDM (*p* = 0.182), post-term deliveries (*p* = 0.480) or stillbirth (*p* = 0.251). The overall incidence of SGA was increased (*p* < 0.001), and that of LGA decreased (*p* < 0.001), in the bariatric group. Five-minute Apgar scores < 7 were detected more often (*p* < 0.001) in the bariatric group (Table 1).

**Table 1:** Maternal, pregnancy and newborn characteristics in the study groups

	Bariatric group *n* = 314	Non-bariatric group *n* = 750,019	*p* value
Age	33.9 (± 5.0)	30.2 (± 5.4)	< 0.001
BMI	33.6 (± 6.4)^a^	24.4 (± 4.8)^b^	< 0.001
Smoking during pregnancy	81 (25.8)	112327 (15.0)	< 0.001
Primiparity	126 (40.1)	313049 (41.7)	< 0.001
Multiparity (> 3 deliveries)	43 (13.7)	74114 (9.9)	< 0.001
Miscarriages (≥ 1)	95 (30.3)	160108 (21.3)^c^	< 0.001
IVF	22 (7.0)	17661 (2.4)	< 0.001
Multiple births	5 (1.6)	10869 (1.4)	0.811
PIH	18 (5.7)	21504 (2.9)	0.002
Pre-eclampsia	5 (1.6)	14892 (2)	0.618
GDM	51 (16.2)	89348 (11.9)	0.018
Insulin treatment for GDM	9 (2.9)	13886 (1.9)	0.182
Preterm contractions	22 (7.0)	32807 (4.4)	0.023
Premature delivery (< 37 weeks)	28 (8.9)	38621 (5.1)	0.003
Post-term delivery (> 42 weeks)	12 (3.8)	34961 (4.7)	0.480
Induction of labor	106 (33.89)	146019 (19.5)	< 0.001
Vaginal birth	218 (69.4)	558185 (74.4)	< 0.001
Planned CS	39 (12.4)	49196 (6.6)	< 0.001
Unplanned CS	42 (13.4)	73892 (9.9)	0.036
Stillbirth	0 (0.0)	2199 (0.3)	0.251
SGA	24 (7.6)	24418 (3.3)	< 0.001
LGA	6 (1.9)	20256 (2.7)	< 0.001
Apgar < 7 (5 min)	17 (5.87)	15352 (2.40)	< 0.001
Apgar 9–10 (5 min)	245 (78.0)	564655 (75.3)	

The data is presented as mean (±SD) or number (%)

*BMI* body mass index,* IVF* in vitro fertilization,* PIH* pregnancy induced hypertension,* GDM* gestational diabetes mellitus,* CS* cesarean section,* SGA* small for gestational age,* LGA* large for gestational age

In the BMI-specific analysis, we detected that women in the bariatric group were older (*p* < 0.001) in all groups, excluding the group with BMI ≤ 24.9 kg/m^2^. Smoking was significantly more common among bariatric group women with BMI ≤ 24.9 kg/m^2^ (*p* < 0.001) and BMI 25.0–29.9 kg/m^2^ (*p* = 0.002). No difference was detected in the incidence of primiparity, multiparity and multiple births. Bariatric group women with BMI 30–34.9 kg/m^2^ (*p* = 0.001) and BMI ≥ 35 kg/m^2^ (*p* < 0.001) had a significantly increased need for IVF treatment, and the risk for miscarriage significantly increased with BMI ≥ 35 kg/m^2^ (*p* = 0.047) (Table 2).

**Table 2:** Comparison of pregnancy and newborn outcomes in BMI-specific groups

	BMI < 25.0 kg/m^2^			BMI 25.0–29.9 kg/m^2^			BMI 30.0–34.9 kg/m^2^			BMI ≥ 35 kg/m^2^		
	Bariatric group *n* = 22	Non-bariatric group *n* = 471,123	*p* value	Bariatric group *n* = 70	Non-bariatric group *n* = 156,702	*p* value	Bariatric group *n* = 97	Non-bariatric group *n* = 58,283	*p* value	Bariatric group *n* = 114	Non-bariatric group *n* = 28,481	*p* value
Age	31.2 (6.1)	29.9 (5.3)	0.32	34.2 (5.1)	30.7 (5.1)	< 0.001	33.7 (4.6)	30.7 (5.5)	< 0.001	34.5 (5.0)	30.7 (5.4)	< 0.001
Smoking during pregnancy	9 (40.9)	6567 (13.9)	< 0.001	21(30.0)	25236 (16.1)	0.002	26 (26.8)	11238 (19.3)	0.061	24 (21.1)	5977 (20.1)	0.986
Primiparity	8 (36.4)	208537 (44.3)	0.456	29 (41.4)	58987 (37.6)	0.513	42 (43.3)	20655 (35.4)	0.106	44 (38.6)	9684 (34.0)	0.301
Multiparity (> 3 deliveries)	1 (4.6)	40881 (8.7)	0.491	7 (10.0)	18132 (11.6)	0.681	11 (11.3)	7802 (13.4)	0.554	24 (21.1)	4157 (14.6)	0.051
Miscarriages (≥ 1)	4 (18.2)	95057 (20.2)	0.816	23 (32.9)	36285 (23.2)	0.054	26 (26.8)	14377 (24.7)	0.626	39 (34.2)	7416 (26.0)	0.047
IVF	1 (4.6)	11 441 (2.4)	0.519	3 (4.3)	3744 (2.4)	0.299	7 (7.2)	1319 (2.3)	0.001	11 (9.7)	396 (1.4)	< 0.001
Multiple births	0 (0)	6506 (1.4)	0.579	0 (0)	2370 (1.5)	0.300	2 (2.1)	933 (1.6)	0.718	2 (1.8)	444 (1.6)	0.867
PIH	1 (4.6)	9852 (2.1)	0.421	4 (5.7)	5732 (3.7)	0.360	4 (4.1)	3354 (5.8)	0.491	9 (7.9)	2243 (7.9)	0.994
Pre-eclampsia	0 (0)	7852 (1.7)	0.541	1 (1.4)	3681 (2.4)	0.611	1 (1.0)	1803 (3.1)	0.241	3 (2.6)	1192 (4.2)	0.408
GDM	5 (22.7)	28668 (6.1)	0.001	32 (45.7)	29461 (18.8)	< 0.001	46 (47.4)	17492 (30.0)	< 0.001	58 (50.9)	12391 (43.5)	0.113
Insulin treatment for GDM	0 (0)	3460 (0.7)	0.687	0 (0)	4126 (2.6)	0.169	5 (5.2)	3242 (5.6)	0.861	4 (3.5)	2901 (10.2)	0.019
Preterm contractions	0 (0)	8442 (1.8)	0.526	1 (1.4)	2377 (1.5)	0.952	4 (4.1)	904 (1.6)	0.041	2 (1.8)	423 (1.5)	0.813
Premature delivery (< 37 weeks)	3 (13.6)	22984 (4.9)	0.057	7 (10.0)	8021 (5.1)	0.064	8 (8.3)	3334 (5.7)	0.284	7 (6.1)	1845 (6.5)	0.884
Post-term delivery (> 42 weeks)	0 (0)	20557 (4.4)	0.316	1 (1.4)	8056 (5.1)	0.160	3 (3.1)	2985 (5.1)	0.365	8 (7.0)	1577 (5.5)	0.491
Induction of labour	4 (18.2)	78458 (16.7)	0.847	22 (31.4)	35306 (22.5)	0.075	34 (35.1)	16610 (28.5)	0.153	45 (39.5)	10196 (35.8)	0.414
Vaginal birth	14 (63.5)	404318 (85.8)	0.003	50 (71.4)	127526 (81.4)	0.032	79 (81.4)	45343 (77.8)	0.388	87 (76.3)	20743 (72.8)	0.404
Planned CS	5 (22.7)	26842 (5.7)	< 0.001	11 (15.7)	11524 (7.4)	0.007	9 (9.3)	5062 (8.7)	0.836	11 (9.7)	3106 (10.9)	0.667
Unplanned CS	3 (13.6)	39939 (8.5)	0.385	9 (12.9)	17647 (11.3)	0.673	9 (9.3)	7877 (13.5)	0.223	16 (14.0)	4631 (16.3)	0.520
Stillbirth	0 (0)	1207 (0.3)	0.813	0 (0)	517 (0.3)	0.633	0 (0)	188 (0.3)	0.576	0 (0)	120 (0.4)	0.489
SGA	1 (4.6)	16910 (3.5)	0.799	5 (7.1)	4465 (2.8)	0.028	9 (9.3)	1642 (2.8)	< 0.001	9 (7.8)	836 (2.9)	0.002
LGA	0 (0)	8600 (1.8)	0.525	1 (1.4)	5811 (3.7)	0.321	1 (1.0)	303 (0.5)	0.481	4 (3.5)	2166 (7.5)	0.103
Apgar 0–6 (5 min)	2 (9.1)	9319 (2.0)	0.015	3 (4.3)	3754 (2.4)	0.289	3 (3.1)	1553 (2.6)	0.786	7 (6.1)	1051 (3.6)	0.161
Apgar 9–10 (5 min)	17 (77.3)	371199 (77.7)	0.961	59 (84.3)	125062 (78.6)	0.247	81 (82.7)	46332 (78.2)	0.289	86 (74.8)	22159 (76.6)	0.648

*IVF* in vitro fertilization,* PIH* pregnancy induced hypertension,* GDM* gestational diabetes mellitus *CS* cesarean section,* SGA* small for gestational age,* LGA* large for gestational age

The BMI-specific analysis showed no difference in the incidence of PIH and pre-eclampsia, but the incidence of GDM was significantly higher among all bariatric group women with BMI < 35 kg/m^2^ (all *p* < 0.001). However, no difference was detected in the need for insulin treatment. An increased incidence of premature contractions was only detected in one BMI group (BMI 30–34.90 kg/m^2^, *p* = 0.041), and a non-significant increase in premature deliveries was detected in the ≤ 24.9 kg/m^2^ (*p* = 0.057) and 25–30 kg/m^2^ (*p* = 0.064) BMI groups. The BMI-specific comparison showed no difference in the incidence of labour induction. In both < 30 kg/m^2^ BMI groups, the bariatric group incidences were significantly lower (*p* = 0.003, *p* = 0.032) for a vaginal birth and significantly higher for planned CS (*p* < 0.001, *p* = 0.007). The was no difference in the incidence of unplanned CS (Table 2).

Excluding the bariatric group with BMI ≤ 24.9 kg/m^2^, the incidence of SGA was higher in other bariatric groups (*p* = 0.028, *p* < 0.001 and *p* = 0.002, respectively), but no difference was detected in the incidence of LGA. Even though the incidence of five-minute Apgar scores < 7 was higher (*p* < 0.001) in the bariatric group, a significant difference (*p* = 0.015) was only detected in one BMI group (BMI ≤ 24.9 kg/m^2^) (Table 2).

## Discussion

The findings of this study provide a good insight into pregnancies and deliveries after BS. Consistent with the results of previous studies, women in the bariatric group were older [11] and had more previous deliveries [9]. These findings are assumably associated with each other. However, the differences in primi- and multiparity disappeared when comparing BMI-specific groups. Even after BS, women in the bariatric group had significantly higher BMI. Residual obesity, and even weight gain a few years after BS, was also seen in the study by Kushner et al. [12].

It has been described that obese people tend to be more prone to smoking [13], and that cessation of smoking may even increase adiposity [14]. The incidence of smoking in the bariatric group was higher than in other studies [9], but, interestingly, this was only seen among women with BMI < 30 kg/m^2^. A possible association with lower post-BS weight is, however, difficult to show. Since smoking was self-reported, bias cannot be excluded. However, differences between BMI groups are unlikely.

The improved prognosis of pregnancies after BS has been previously described [15] and is rational when comparing pregnancies before and after BS [16]. An important question concerns whether the prognosis is just associated with post-BS weight and if surgery-associated risks exist.

Even though BS has a positive effect on fertility [17], increasing age [18] and obesity itself [3] impair it.

A previous study [17] reported that the only predictor of fertility after BS is BMI. Accordingly, we detected an increased need for IVF only among women with post-BS BMI ≥ 30 kg/m^2^. Even though we did not study the results of IVF treatments, obesity is expected to have a negative impact on those results. An increased incidence of miscarriage was detected among women with post-BS BMI > 35 kg/m^2^. In a previous study, the association with BS was not evaluated and is assumed to be related to obesity itself [3].

The risk of hypertensive disorders [3, 19] is increased by being overweight. Our study also showed a higher overall incidence of PIH in the BS-group, but no difference was detected in the BMI-specific comparison. Compared with the results of Bennett et al. [20], we did not detect an increased incidence of pre-eclampsia.

As excessive fat deposition is known to impair glucose metabolism [3], we detected a generally increased incidence of GDM in the bariatric group. Excluding post-BS women with BMI ≥ 35 kg/m^2^, the incidence was significantly higher in the BMI-specific comparisons (all *p* = 0.001). The incidence of GDM was also high among non-bariatric women with BMI ≥ 35 kg/m^2^ and may explain the equal incidences between groups.

Even though the metabolic changes after BS have been well described [21], it is worth noting that the diagnostics of GDM in the bariatric and non-bariatric groups are different in Finland. Instead of the normally used 75 g glucose tolerance test, home monitoring of sugar levels is used in post-BS women to avoid dumping syndrome. According to previous studies, up to 40–50% of post-BS patients suffer from dumping syndrome [22]. However, no specific guidelines on GDM screening and treatment after BS exist. We did not detect an increased need for insulin treatment, but this may be explained to some extent by the small number of cases. The changes in glucose metabolism have been shown to be associated with the type of BS [23], but that was not recorded in this study.

Consistent with previous studies [3, 8], the incidence of pre-term delivery was increased after BS. Some studies have reported an increased incidence partly explained by obesity-related pregnancy complications and the need for iatrogenic pre-term delivery [3], while some studies have found no difference in the incidence of iatrogenic pre-term deliveries after BS [23]. In our study, a non-significantly increased incidence of pre-term deliveries was only seen in the two BMl categories < 30 kg/m^2^. The proportion of iatrogenic prematurity could not be analysed retrospectively, but the incidence of the most common indications, namely PIH, pre-eclampsia and SGA, was not increased. The risk of prematurity increases along with maternal age [24], and therefore the possible additional risk caused by BS is difficult to estimate. Even though opposing results have been reported [11], our study did not show any difference in the incidence of post-term deliveries.

Consistent with our results, an increased need for labour induction among obese women has been reported [3, 25]. However, a reduction in inductions after BS was reported when compared with BMI-matched controls [10]. This may be associated with the increased incidence of pre-term deliveries and a reported decreased incidence of post-term deliveries. Our BMI-specific analysis did not reveal differences between BMI groups. It is also noteworthy that the incidence of labour inductions was increased, even though common indications for induction (e.g., hypertensive disorders and diabetes) were decreased. Could it be that we easily tend to intervene in the natural course of these post-BS pregnancies?

The association between BS and CS is contradictory [26], and some studies consider BS to be an independent risk factor for CS [27]. In our study, the overall incidence of both planned and unplanned CS was significantly higher in the bariatric group. The BMI-specific comparison revealed an increased incidence of planned CS only among post-BS women with BMI < 30 kg/m^2^. This result is difficult to explain and may be biased by the small BMI groups and the small number of CS cases. Lapolla et al. [28] reported an increased CS incidence only when compared with normal-weight women, but when compared with non-BS obese women in the same study, the incidence of CS was reduced. Other studies have also reported a reduced incidence of CS after BS when compared with BMI-matched controls [11]. The independent impact of BS is, however, difficult to evaluate, since the risk of CS also increases with age, overweight and obesity [29].

The previously reported increased risk of stillbirth [3] was not detected in our study. This risk has been explained by the impaired detection of fetal macrosomia, possible abnormalities and reduced fetal movements [3]. We did not find any difference in the incidence of LGA, and despite the significantly increased incidence of GDM, the incidence of SGA was significantly higher and detected in all BMI groups with BMI ≥ 25 kg/m^2^. Our results are supported by earlier studies [8, 21] and assumed to be explained by nutritional deficiency and malnutrition following BS, especially after malabsorptive surgeries [11], although associations with the type of surgery were not analysed in this study. The impact of smoking on the incidence of SGA cannot be reliably excluded.

We detected a higher incidence of five-minute Apgar scores of 0–6 in the bariatric group with a BMI < 25.0 kg/m^2^. Since the sample sizes in this BMI group were small, the incidence of planned CS was the highest and no difference was detected in the incidences of SGA, LGA, PIH or pre-eclampsia, the small number of cases is likely to have caused bias in the results. Lower Apgar scores were not reported in the nationwide register-based study by Kjær et al. [26].

The detailed analysis of pregnancies and high-quality register-based data can be considered as strengths of this study. Since, in Finland, all deliveries and the majority of BS are taken care of in public hospitals [6], all data concerning pregnancies, deliveries and BS are reported to the Finnish Institute of Health and Welfare. This is also done in possible cases of BS in private clinics. Rather than comparing women with controls matched with pre-surgery weight [23], we aimed to show the prognosis of post-BS women compared with their BMI-matched non-bariatric controls. We consider this to be another strength of this study.

A register-based study is limited to the use of recorded variables, and minor differences in the use of diagnosis between persons, departments and institutions cannot be excluded. Weight gain or possibly ongoing post-BS weight loss during pregnancy was not recorded. In addition, the data on BS techniques were not available for us. A previous study has shown that malabsorptive technique is associated with poorer pregnancy outcomes [31], but when restrictive and combination technique such as gastric bypass was compared, no significant differences in the pregnancy outcomes were found [30]. The time between the surgery and the following pregnancy will be discussed in a separate article. These factors can be considered as limitations of this study. Bias caused by the small number of cases in some variables and BMI-specific comparisons cannot be excluded.

This study provides important data on the impact of BS in future pregnancies and deliveries that can be used when counselling women with family planning before or after BS. As the pre-BMI data was not available for us and the post-BS improvements in obstetric outcomes have already been described [15, 16], we especially aimed to study the differences in obstetric outcomes between operated and non-operated BMI-matched women. Based on this study, and considering all the benefits and risks of BS, we consider the procedure to be a safe and advisable treatment for obesity among fertile-age women. We conclude that the prognosis of pregnancy is highly associated with achieved weight loss, and the need for assisted fertilisation remains increased among women with residual obesity. According to our results, the risk for hypertensive disorders is comparable with BMI-matched controls, but the risk for GDM is significantly increased. Still, the GDM-associated risk of LGA is minimal after BS. Despite the good prognosis of post-BS pregnancies, surveillance by an obstetrics specialist is important for securing the optimal outcome for both the mother and the baby [32].

## Data Availability

The authors confirm that the data supporting the findings of this study are available within the article and its supplementary material. Raw data that support the findings of this study are available from the corresponding author, upon reasonable request.
